# Physical Activity, TV Watching Time, Sleeping, and Risk of Obesity and Hyperglycemia in the Offspring of Mothers with Gestational Diabetes Mellitus

**DOI:** 10.1038/srep41115

**Published:** 2017-01-25

**Authors:** Tao Zhang, Peng Wang, Huikun Liu, Leishen Wang, Weiqin Li, Junhong Leng, Nan Li, Shuang Zhang, Lu Qi, Jaakko Tuomilehto, Zhijie Yu, Xilin Yang, Gang Hu

**Affiliations:** 1 Tianjin Women’s and Children’s Health Center, 96 Guizhou Road, Heping Districts, Tianjin, China; 2 Chronic Disease Epidemiology Laboratory, Pennington Biomedical Research Center, Baton Rouge, LA, USA; 3 Department of Epidemiology, School of Public Health and Tropical Medicine, Tulane University, New Orleans, LA, USA; 4 Dasman Diabetes Institute, Dasman, Kuwait; 5 Population Cancer Research Program and Department of Pediatrics, Dalhousie University, Halifax, Canada; 6 Department of Epidemiology, School of Public Health, Tianjin Medical University, Tianjin, China

## Abstract

We investigated the association of physical activity, TV watching time, sleeping time with the risks of obesity and hyperglycemia among 1263 offspring aged 1–5 years of mothers with gestational diabetes (GDM) in a cross-sectional study. Logistic regression models were used to obtain the odd ratios (ORs) (95% confidence intervals [CI]) of childhood obesity and hyperglycemia associated with different levels of indoor activity, outdoor activity, TV watching, and sleeping time. The multivariable-adjusted ORs of obesity based on different levels of TV watching time (0, <1.0, and ≥1.0 hour/day) were 1.00, 1.21 (95% CI 0.72–2.05), and 2.20 (95% CI 1.33–3.63) (P_trend_ = 0.003), respectively. The multivariable-adjusted ORs of hyperglycemia based on different levels of indoor activity (<5.0, 5.0–6.9, and ≥7.0 hours/day) were 1.00, 0.74 (95% CI 0.45–1.21), and 0.49 (95% CI 0.28–0.84) (P_trend_ = 0.034), respectively. The multivariable-adjusted ORs of hyperglycemia associated with different levels of sleeping time (<11.0, 11.0–11.9, and ≥12.0 hours/day) were 1.00, 0.67 (95% CI 0.42–1.05), and 0.39 (95% CI 0.23–0.67) (P_trend_ = 0.003), respectively. The present study indicated a positive association of TV watching with the risk of obesity, and an inverse association of either indoor activity or sleeping time with the risk of hyperglycemia among offspring born to GDM mothers in Tianjin, China.

It is widely acknowledged that obesity has become a leading public health problem all over the world^1^,^2^. Especially the prevalence of obesity among children and adolescents which is increasing at an alarming rate is critical^3^, not only because obese children tend to become obese adults^4^,^5^, but also they are more likely to suffer from hyperlipidemia, hypertension, insulin resistance, respiratory problems and orthopedic complications than non-obese children^6^. In China, with the rapid economic development and urbanization, Chinese people’s lifestyle changed tremendously in recent years, which resulted in an increasing prevalence of obesity, and the prevalence of overweight/obesity in the urban population has reached the same level as that of some developed countries^7^. The International Study of Childhood Obesity, Lifestyle and the Environment (ISCOLE) has shown that the prevalence of obesity among Chinese children is far greater than the average of 12 countries^8^,^9^. Many studies showed evidence that the increased prevalence of childhood obesity has been attributed largely to behavioral and environment factors^10^. Thus identifying risk factors at early time and developing effective intervention programs might be a significant strategy for obesity prevention.

Several studies have found that decreases in physical activity and increases in sedentary behavior, such as TV viewing and video/computer use, blame on the secular trends in obesity among children^8^,^9^. A recent study found that short sleep duration was associated with a higher risk of childhood obesity^11^, however, another cohort study found that sleep restriction did not increase future risk of obesity^12^. Few studies assessed this association among children less than 5 years old, but the results remain elusive^13^. Understanding this question is very important because eating and physical activity habits that contribute to later obesity become established during formative pre-school years (2–6 years old) and these habits are more malleable at this time than in later childhood^14^. Moreover, the associations of physical activity, sedentary behavior, sleeping time with the risk of childhood obesity among the offspring of gestational diabetes mellitus (GDM) mothers are not clear. It has been shown that offspring of mothers exposed to GDM were at increased risk of neonatal adiposity and childhood obesity^15^,^16^,^17^,^18^, thus assessing different types of physical activity with the risk of obesity among offspring of mothers exposed to GDM is important, which might improve early intervention among these children. The purpose of this study is to investigate the association of physical activity, TV watching time, and sleeping time with the risks of obesity, central obesity and hyperglycemia among the offspring aged 1–5 years of GDM mothers who participated in the Tianjin Gestational Diabetes Mellitus Prevention Program (TGDMPP)^19^.

## Results

Of 1263 children, the prevalence of obesity, central obesity, and hyperglycemia were 10.1%, 10.0%, and 10.0%, respectively. General characteristics of both mothers and children according to different childhood obesity, center obesity and hyperglycemia status are presented in Table 1.

The multivariable-adjusted ORs (model 1) of obesity associated with different levels of childhood TV watching time (0, <1.0, and ≥1 hour per day) were 1.00, 1.25 (95% CI 0.74–2.09), and 2.31 (95% CI 1.41–3.80) (P_trend_ = 0.001) (Table 2). After further adjustment for indoor activity, outdoor activity and sleeping time (multivariable-adjusted model 2), this positive association was still significant (P_trend_ = 0.003). There were no associations of indoor activity, outdoor activity, and sleeping time with the risk of childhood obesity.

The multivariable-adjusted ORs (model 1) of central obesity associated with different levels of childhood TV watching time (0, <1.0, ≥1.0 hour per day) were 1.00, 1.28 (95% CI 0.78–2.08), and 1.76 (95% CI 1.08–2.88) (P_trend_ = 0.071) (Table 3). After additional adjustment for indoor activity, outdoor activity and sleeping time (multivariable-adjusted model 2), children who watched ≥1.0 hour TV per day had 73% (95% 1.05–2.84) high risk of central obesity compared with those who did not watch TV. There were no associations of indoor activity, outdoor activity, and sleeping time with the risk of central obesity.

Table 4 showed the associations of different levels of indoor activity, outdoor activity, TV watching time and sleeping time with the risk of hyperglycemia. The multivariable-adjusted ORs (model 1) of hyperglycemia based on different levels of indoor activity (<5.0, 5.0–6.9, and ≥7.0 hours per day) were 1.00, 0.71 (95% CI 0.43–1.17), and 0.52 (95% CI 0.31–0.88) (P_trend_ = 0.052), respectively. This association became stronger after adjustment for outdoor activity, TV watching time and sleeping time (multivariable-adjusted model 2) (P_trend_ = 0.034). The multivariable-adjusted ORs (model 1) of hyperglycemia associated with different levels of sleeping time (<11.0, 11.0–11.9, and ≥12.0 hours per day) were 1.00, 0.68 (95% CI 0.43–1.06), and 0.42 (95% CI 0.25–0.71) (P_trend_ = 0.005). This inverse association was still significant after additional adjustment for indoor activity, outdoor activity, and TV watching time (multivariable-adjusted model 2) (P_trend_ = 0.003). However, there were no associations of outdoor activity and TV watching time with the risk of hyperglycemia.

When each type of physical activity was examined as a continuous variable, multivariate-adjusted ORs (model 2) of obesity and central obesity associated with each 1 hour/day increase in TV watching time were 1.37 (95% CI, 1.16–1.63), and 1.24 (95% CI, 1.04–1.47) (Tables 2 and 3), respectively. Multivariate-adjusted ORs (model 2) of hyperglycemia were 0.90 (95% CI, 0.82–1.00) for each 1 hour/day increase in indoor activity, and 0.78 (95% CI, 0.66–0.91) for each 1 hour/day increase in sleeping time (Table 4).

## Discussion

The present study indicated a positive association of TV watching time with the risk of obesity and central obesity, and an inverse association of either indoor activity or sleeping time with the risk of hyperglycemia among offspring born to GDM mothers in Tianjin.

To our knowledge, this is the first study assessing the association of indoor activity, outdoor activity, TV watching time, and sleeping time with the risks of obesity, central obesity and hyperglycemia among preschool offspring of mothers with gestational diabetes mellitus. Several studies have shown that diet, physical activity, and behavioral modifications are effective in reducing childhood obesity^20^,^21^, however, obesity is difficult to reverse in older children and adults^14^. Thus developing effective prevention and intervention programs for the children at formative pre-school years (2–6 years old) might be an important step in combating the childhood obesity epidemic.

Several studies have assessed the association between TV watching time and obesity risk among school-aged children^22^. A cross-sectional study conducted by Dennison, B. A. *et al*. found that TV viewing time and TV in the child’s bedroom were associated with overweight risk in preschool children^23^. Another cross-sectional study from 34 countries found that television viewing times were higher in overweight compared to normal weight youth at 10–16 years old^24^. The International Study of Childhood Obesity, Lifestyle and the Environment (ISCOLE) found a positive association between TV watching time and the risk of obesity among 6026 children at 9–11 years of age in 12 countries^8^. It has been suggested that TV watching as an aspect of sedentary behavior appears to be more strongly related to obesity than other sedentary behaviors^25^, because TV watching has a low metabolic rate and may replace other physical activities with higher energy demands. In addition, children usually eat something or take snacks with increased caloric intake when they watch TV. Very few studies have examined the association between TV watching and childhood obesity among children under 3–5 years old in China. Thus it was unclear how early in life TV viewing in children began to express the risk of childhood obesity. Childhood obesity experts suggest to discourage television viewing in children under two years of age but 14% of children aged 6 to 23 months watch 2 or more hours/day of media^26^ and TV viewing is common in this age group. In the present study, we found that 52% of children under 2 years old watched television, and 82% of children at 4 years old watched TV. Children who watched ≥1.0 hour TV per day had 129% and 78% high risk of obesity and central obesity compared with those who did not watch TV.

Some studies have assessed the association between physical activity and the risk of childhood obesity, and the results are controversial. Several studies provided supportive information of an inverse association^8^; however, others have reported no association^27^,^28^. In the present study, we found an inverse association of indoor activity and the risk of hyperglycemia, and no association of indoor activity and outdoor activity with the risk of childhood obesity and central obesity. There are several reasons for the difference in associations across studies. First, the information of physical activity was obtained based on parents’ reports, especially for young children, which might produce recall-bias. Second, the intensity of physical activity was not generally assessed by questionnaire which is known to be important especially for young children who may alternatively take part in the types of less vigorous-intensity activities.

A 12-country study has found a significant association between short sleep duration and obesity risk in children at 9–11 years of age^8^. One recent review and one meta-analysis have also shown a clear association between short sleep duration and an increased risk of childhood obesity, however, very few studies in this meta-analysis included children less than 5 years of age^29^,^30^. The present study found an inverse association between sleeping time and the risk of hyperglycemia, which supported the previous finding^31^. However, the present study did not find any associations between sleeping time and the risks of childhood obesity and central obesity. The reason may be that the sleep duration definition is conservative and the sleeping time is different between younger and older children. Thus large studies including children less than and more than 5 years old are needed.

One recent study based on the US population found that waist circumference and BMI could equally estimate total fat and visceral fat in children^32^. It has been hypothesized that Asian adults have a higher percentage of body fat at a given BMI value compared with European populations^33^. The present study did not find any different associations between obesity risk and central obesity risk with different types of physical activity.

This study has several strengths and limitations that warrant discussions. First, we extended to study the association of different types of physical activity and the risks of obesity, central obesity and hyperglycemia among children under 5 years old. Second, children’s weight, height, waist circumference and fasting glucose were measured by trained health workers rather than by parental report. There are several limitations in this study. First, using a parent-reported questionnaire to assess children’s indoor activity, outdoor activity, TV watching time, and sleeping time is crude and imprecise because the physical activity in preschool-aged children consists of short intermittent peaks of activity with frequent rest periods, which is different from the physical activity in adults characterized by constant or systematic periods. Although the performance of this questionnaire has been used in a longitudinal study in the same area of Tianjin^34^,^35^,^36^, studies of accurately measuring children’s physical activity such as using 24-hour accelerometers are needed. Another limitation of this study is that only part of children aged more than 24 months were asked diet habits by a parent self-administered food frequency questionnaire (FFQ). Thus we cannot control for dietary intake on the association of physical activity, TV watching time, and sleeping time with the risks of childhood obesity and hyperglycemia. We cannot completely exclude the effects of residual confounding resulting from measurement error in the assessment of confounding factors or some unmeasured factors. As our study was conducted among the offspring of GDM, we could not compare the influence of physical activity, sedentary behavior, sleeping time with the risk of childhood obesity between children from non-GDM mothers and those from GDM mothers. Several studies have shown that offspring of mothers exposed to GDM were at increased risk of neonatal adiposity and childhood obesity, however, the interaction of physical activity, sedentary behavior, sleeping time and maternal GDM with the risk of childhood obesity is not clear, thus further studies are needed to answer this question.

In summary, this study demonstrated that more time spending on TV watching appears to be associated with a higher risk of obesity and central obesity among 1–5 years old offspring of GDM mothers. The effect of short sleep duration on hyperglycemia risk in these children appears to be independent of other risk factors.

## Methods

### Tianjin Gestational Diabetes Mellitus Prevention Program

Tianjin is the fourth largest city in Northern China. All pregnant women who live in six urban districts have participated in the universal screening for GDM since 1999^37^ by using the World Health Organization (WHO)’s GDM criteria^38^. From December 1998 to December 2009, a total of 128,125 pregnant women took part in the GDM screening program and 6,247 were diagnosed with GDM^19^. The average proportion of screened pregnancies was over 91% during 1999–2009^37^.

### Study samples

The sampling methods have been described previously in detail^19^. A total of 4,644 pregnant women who were diagnosed with GDM from 2005 to 2009 and their children in six urban districts were invited to participate in a baseline survey for the TGDMPP. Finally, 1263 GDM women and their children had completed the baseline survey for the TGDMPP from August 2009 to July 2011 (participation rate 27%)^19^,^39^,^40^,^41^,^42^,^43^. The study followed the guidelines of the Helsinki Declaration, and was approved by the Human Subjects Committee of the Tianjin Women’s and Children’s Health Center, informed consent was obtained for each participant.

### Examinations

At baseline survey, all GDM mothers and their children completed a self-administered questionnaire and underwent a physical examination that included anthropometric and blood pressure measurements, immediate blood glucose, a 2-hour glucose 75 g OGTT (mothers only), and a fasting blood draw at the Tianjin Women’s and Children’s Health Center. Health workers from the Tianjin Women’s and Children’s Health Center collected and checked the completed questionnaire and also finished measurements. All health workers were intensively trained in meetings and in practical sessions. The questionnaire included questions on the mother’s socio-demographics (age, marital status, education, income, and occupation), history of GDM (values of fasting and 2-hour glucose in the OGTT at 26–30 gestational weeks from the Tianjin Women’s and Children’s Health Center GDM diagnosis and treatment register system), family history of chronic diseases, medical history (hypertension, diabetes, and hypercholesterolemia), and pregnancy outcomes (pre-pregnancy weight, weight gain during pregnancy, and number of children)^42^. We also asked the GDM children’s parents in advance to bring the child’s birth certificate and to fill in a self-administered questionnaire about the child’s birth date, sex, gestational weeks of birth, birth weight, birth recumbent length, Apgar score (above questions related to birth were copied from birth certificate), the mode and duration of infant feeding (exclusive breast feeding, mixed breast and formula feeding, and exclusive formula feeding), health characteristics (history of illness status and current health status), dietary habits (usual habits of eating breakfast, lunch, and dinner, usual frequency of intake of vegetables, fruits, sugar-sweetened beverages, and fast food), physical activity habits (duration of indoor activity, outdoor activity), usual sleeping time, and TV or computer watching time.

To determine indoor activity time, we asked the question “How many hours per day did your child play indoors during the past year ? ” and grouped it into 3 categories: <5.0, 5–6.9, and ≥7.0 hours per day. We asked the question “How many hours per day did your child play outdoors during the past year in each season separately ? ” and calculated daily mean time (sum of different seasons/4) and classified it into three categories: <1.5, 1.5–1.9, and ≥2.0 hours per day. TV watching time was assessed using the question “How many hours per day did your child watch TV during the past year ? ” and the response categories were 0, <1.0, and ≥1.0 hour per day. We also asked the question “How many hours per day did your child sleep during the past year ? ” and grouped it into 3 categories: <11.0, 11.0–11.9, and ≥12.0 hours per day. We have done classification for the indoor/outdoor activity time, TV watching time, and sleeping time based on the distributions and mean values of each activity, and then used tertiles or quartiles to assess different activities. We also investigated the association of indoor activity, outdoor activity, TV watching time, and sleeping time as continuous variables with the risks of childhood obesity, central obesity and hyperglycemia.

Children’s body weight was measured with a beam balance scale with participants wearing light indoor clothing without shoes, and height was measured by a stadiometer. Waist circumference was measured midway between the 10^th^ rib and the top of the iliac crest. Weight was measured to the nearest 0.1 kg, height and waist circumference to the nearest 0.1 cm. Body mass index (BMI) was calculated by dividing weight in kilograms by the square of height in meters. BMI for age Z score was calculated, and we took a more recent expert committee recommendation^44^ that obesity was defined as a BMI more than the 95^th^ percentiles (≥1.645 Z score) for age and gender specific distribution using WHO growth ref.^45^. Central obesity was defined as more than the 90^th^ percentiles for age and gender specific distribution of waist circumference^47^. About 150 μl of at least 6-hour fasting capillary whole blood specimen was collected and the fasting time was recorded. Blood glucose level was immediately measured on an automatic analyzer (Biosen C-line; EKF Diagnostic GmbH, London, U.K.)^48^. Hyperglycemia was defined as more than the 90^th^ percentiles of fasting glucose.

### Statistical analyses

The general characteristics of both mothers and children according to different childhood obesity, central obesity and hyperglycemia status were compared using independent-samples T-Test for continuous variables and chi-square tests for categorical variables. Logistic regression was used to estimate the odd ratios (ORs) and 95% confidence intervals (CIs) of childhood obesity, central obesity and hyperglycemia associated with different levels of indoor activity, outdoor activity, TV watching time, and sleeping time. We set up two models: Model 1, adjusted for variables included maternal age, weight gain during pregnancy, education, occupation, family income, and child age, sex, gestational age, and mode of infant feeding; Model 2, adjusted for variables in Model 1 and, additionally, for TV watching time, and sleeping time (in physical activity analysis), indoor activity, outdoor activity and sleeping time (in TV watching time analysis), indoor activity, outdoor activity and TV watching time (in sleeping time analysis). All statistical analyses were performed with PASW for Windows, version 23.0 (Statistics 23, SPSS, IBM, USA).

## Additional Information

**How to cite this article**: Zhang, T. *et al*. Physical Activity, TV Watching Time, Sleeping, and Risk of Obesity and Hyperglycemia in the Offspring of Mothers with Gestational Diabetes Mellitus. *Sci. Rep.* **7**, 41115; doi: 10.1038/srep41115 (2017).

**Publisher's note:** Springer Nature remains neutral with regard to jurisdictional claims in published maps and institutional affiliations.

## Figures and Tables

**Table 1:** Characteristics of study participants among 1263 mother-child pairs according to child’s obesity, center obesity and hyperglycemia status.

	No obesity	Obesity^b^	P values	No central obesity	Central obesity^c^	P values	Normal glucose	Hyperglycemia^d^	P values
No. of subjects	1135	128		1137	126		1137	126	
**Maternal characteristics**									
Maternal age before pregnancy (years)	30.1 (3.5)	29.9 (3.9)	0.56	30.0 (3.4)	30.3 (3.9)	0.43	30.1 (3.5)	30.1 (3.6)	0.91
Gestational age at delivery (weeks)	39.0 (1.5)	39.0 (1.5)	0.76	39.0 (1.5)	38.9 (1.4)	0.58	39.0 (1.5)	39.1 (1.6)	0.65
Gestational weight gain^a^ (%)			0.007			0.08			0.84
Inadequate	14.7	9.4		14.3	12.7		14.1	15.1	
Adequate	31.8	22.7		32.1	19.8		31.0	28.6	
Excessive	53.5	68.0		53.6	67.5		54.9	56.3	
Occupation of mother (%)			0.044			0.10			0.14
Unemployed persons	25.0	23.4		24.5	28.6		25.4	20.6	
Office workers	15.0	23.4		15.3	20.6		16.2	11.9	
Other	60.0	53.1		60.2	50.8		58.4	67.5	
Education of mother (%)			0.012			0.04			0.12
High school and under	2.4	3.9		2.4	4.0		2.6	1.6	
Junior college	18.5	29.7		18.7	27.8		19.6	19.8	
University	71.4	60.9		71.1	63.5		70.7	65.9	
Master above	7.8	5.5		7.8	4.8		7.0	12.7	
Family incomes (yuan/month)	7374	6603	0.23	7343	6865	0.46	7332	6841	0.45
**Child characteristics**									
Age (months)	27.0 (10.6)	28.7 (9.9)	0.087	27.0 (10.4)	28.2 (11.3)	0.22	27.1 (10.5)	28.1 (10.8)	0.34
Boy (%)	51.7	66.4	0.002	53.2	53.2	0.99	51.5	69.0	<0.001
Breast feeding (%)			0.16			0.16			0.97
Exclusive breast feeding	43.9	35.2		43.8	35.7		43.0	42.9	
Mixed feeding	38.9	44.5		38.7	46.8		39.6	38.9	
Exclusive formula feeding	17.2	20.3		17.5	17.5		17.4	18.2	
Indoor activity (%)			0.20			0.35			0.042
<5.0 hours/day	31.8	35.9		31.7	37.3		31.2	41.3	
5.0–6.9 hours/day	32.1	35.9		33.0	27.8		32.5	31.7	
≥7. hours/day	36.1	28.2		35.3	34.9		36.3	27.0	
Outdoor activity (%)			0.73			0.53			0.12
<1.5 hours/day	33.7	30.4		33.2	35.7		33.3	34.9	
1.5–1.9 hours/day	25.5	25.8		25.9	21.4		26.3	18.3	
≥2.0 hours/days	40.8	43.8		40.9	42.9		40.4	46.8	
TV watching time (%)			< 0.001			0.02			0.46
0 hour/day	36.5	25.0		36.1	27.8		35.1	37.3	
<1.0 hour/day	36.7	28.9		36.1	33.3		36.4	31.0	
≥1.0 hours/day	26.8	46.1		27.8	38.9		28.5	31.7	
Sleeping time (%)			0.13			0.34			0.003
<11.0 hours/day	36.3	44.5		36.6	42.0		35.9	49.2	
11.0–11.9 hours/day	30.7	29.7		30.5	31.0		30.6	30.2	
≥12.0 hours/day	33.0	25.8		32.9	27.0		33.5	20.6	

Baseline characteristics represent mean (SD) or percentage.

^a^Gestational weight gain categories: Inadequate (1): <12.5 kg (pre-pregnancy BMI < 18.5 kg/m2), <11.5 kg (BMI 18.5–23.9 kg/m2), <7 kg (BMI 24.0–27.9 kg/m2), and <5 kg (BMI > 28 kg/m2); Adequate (1): 12.5–18 kg (BMI < 18.5 kg/m2), 11.5–16 kg (BMI 18.5–23.9 kg/m2), 7–11.5 kg (BMI 24.0–27.9 kg/m2), and 5–9 kg (BMI > 28 kg/m2); Excessive (1): >18 kg (BMI < 18.5 kg/m2), >16 kg (BMI 18.5–23.9 kg/m2), >11.5 kg (BMI 24.0–27.9 kg/m2), and >9 kg (BMI > 28 kg/m2), according to the Chinese maternal pre-pregnancy BMI classification standard and the 2009 IOM GWG recommendations.

^b^Obesity was defined as a body mass index more than the 95th percentiles (≥1.645 Z score) for age and gender specific distribution using WHO growth reference.

^c^Center obesity was defined as more than the 90th percentiles for age and gender specific distribution of waist circumference.

^d^Hyperglycemia was defined as more than the 90^th^ percentiles of fasting glucose.

**Table 2:** Odd ratios of obesity according to different levels of indoor and outdoor physical activity, TV watching and sleeping time.

	No. of participants	No. of cases	Odd ratios (95% confidence intervals)	
			Model 1*	Model 2†
**Indoor activity categories (hours/day)**				
<5.0	407	46	1.00	1.00
5.0–6.9	410	46	0.98 (0.59–1.64)	1.01 (0.60–1.69)
≥7.0	446	36	0.76 (0.44–1.32)	0.82 (0.47–1.42)
P for trend			0.52	0.66
Indoor activity as a continuous variable (1 hour/day increase)			0.99 (0.90–1.09)	0.97 (0.88–1.08)
**Outdoor activity categories (hours/day)**				
<1.5	422	39	1.00	1.00
1.5–1.9	322	33	0.98 (0.58–1.64)	0.99 (0.59–1.67)
≥2.0	519	56	1.19 (0.75–1.87)	1.13 (0.71–1.79)
P for trend			0.65	0.82
Outdoor activity as a continuous variable (1 hour/day increase)			1.08 (0.88–1.32)	1.09 (0.87–1.38)
**TV watching time categories (hours/day)**				
0	446	32	1.00	1.00
<1.0	453	37	1.25 (0.74–2.09)	1.21 (0.72–2.05)
≥1.0	364	59	2.31 (1.41–3.80)	2.20 (1.33–3.63)
P for trend			0.001	0.003
TV watching time as a continuous variable (1 hour/day increase)			1.39 (1.18–1.65)	1.37 (1.16–1.63)
**Sleeping time categories (hours/day)**				
<11.0	469	57	1.00	1.00
11.0–11.9	386	38	0.97 (0.60–1.56)	0.99 (0.61–1.59)
≥12.0	408	33	0.71 (0.42–1.18)	0.75 (0.44–1.26)
P for trend			0.36	0.49
Sleeping time as a continuous variable (1 hour/day increase)			0.88 (0.74–1.05)	0.89 (0.74–1.06)

Obesity was defined as a body mass index more than the 95th percentiles (≥1.645 Z score) for age and gender specific distribution using WHO growth reference.

*Adjusted for maternal age, pre-pregnancy BMI, weight gain during pregnancy, education, occupation, family income, and child age, sex, gestational age, and mode of infant feeding.

^†^Adjusted for variables in Model 1 and also indoor activity, outdoor activity, TV watching time, and sleeping time, other than the variable in the analyses.

**Table 3:** Odd ratios of central obesity according to different levels of indoor and outdoor physical activity, TV watching and sleeping time.

	No. of participants	No. of cases	Odd ratios (95% confidence intervals)	
			Model 1*	Model 2†
**Indoor activity categories (hours/day)**				
<5.0	407	47	1.00	1.00
5.0–6.9	410	35	0.67 (0.39–1.13)	0.68 (0.40–1.15)
≥7.0	446	44	0.86 (0.51–1.45)	0.89 (0.53–1.51)
P for trend			0.30	0.31
Indoor activity as a continuous variable (1 hour/day increase)			0.98 (0.89–1.08)	0.98 (0.89–1.09)
**Outdoor activity categories (hours/day)**				
<1.5	422	45	1.00	1.00
1.5–1.9	322	27	0.72 (0.43–1.21)	0.75 (0.44–1.26)
≥2.0	519	54	1.01 (0.65–1.56)	1.02 (0.65–1.59)
P for trend			0.36	0.44
Outdoor activity as a continuous variable (1 hour/day increase)			0.98 (0.80–1.20)	0.99 (0.78–1.24)
**TV watching time categories (hours/day)**				
0	446	35	1.00	1.00
<1.0	453	42	1.28 (0.78–2.08)	1.26 (0.77–2.06)
≥1.0	364	49	1.76 (1.08–2.88)	1.73 (1.05–2.84)
P for trend			0.071	0.089
TV watching time as a continuous variable (1 hour/day increase)			1.25 (1.05–1.48)	1.24 (1.04–1.47)
**Sleeping time categories (hours/day)**				
<11.0	469	53	1.00	1.00
11.0–11.9	386	39	1.02 (0.64–1.63)	1.07 (0.67–1.72)
≥12.0	408	34	0.77 (0.46–1.27)	0.83 (0.50–1.39)
P for trend			0.48	0.61
Sleeping time as a continuous variable (1 hour/day increase)			0.91 (0.76–1.08)	0.92 (0.77–1.10)

Center obesity was defined as more than the 90^th^ percentiles for age and gender specific distribution of waist circumference.

*Adjusted for maternal age, pre-pregnancy BMI, weight gain during pregnancy, education, occupation, family income, and child age, sex, gestational age, and mode of infant feeding.

†Adjusted for variables in Model 1 and also indoor activity, outdoor activity, TV watching time, and sleeping time, other than the variable in the analyses.

**Table 4:** Odd ratios of hyperglycemia according to different levels of indoor and outdoor physical activity, TV watching and sleeping time.

	No. of participants	No. of cases	Odd ratios (95% confidence intervals)	
			Model 1*	Model 2†
**Indoor activity categories (hours/day)**				
<5.0	407	52	1.00	1.00
5.0–6.9	410	40	0.71 (0.43–1.17)	0.74 (0.45–1.21)
≥7.0	446	34	0.52 (0.31–0.88)	0.49 (0.28–0.84)
P for trend			0.052	0.034
Indoor activity as a continuous variable (1 hour/day increase)			0.92 (0.84–1.01)	0.90 (0.82–1.00)
**Outdoor activity categories (hours/day)**				
<1.5	422	44	1.00	1.00
1.5–1.9	322	23	0.62 (0.37–1.07)	0.61 (0.35–1.06)
≥2.0	519	59	1.00 (0.65–1.53)	0.89 (0.57–1.39)
P for trend			0.16	0.20
Outdoor activity as a continuous variable (1 hour/day increase)			1.04 (0.84–1.29)	1.12 (0.90–1.41)
**TV watching time categories (hours/day)**				
0	446	47	1.00	1.00
<1.0	453	39	0.80 (0.50–1.28)	0.75 (0.46–1.20)
≥1.0	364	40	1.05 (0.65–1.70)	0.91 (0.56–1.48)
P for trend			0.49	0.47
TV watching time as a continuous variable (1 U hour/day increase)			1.03 (0.86–1.24)	1.00 (0.83–1.20)
**Sleeping time categories (hours/day)**				
<11.0	469	62	1.00	1.00
11.0–11.9	386	38	0.68 (0.43–1.06)	0.67 (0.42–1.05)
≥12.0	408	26	0.42 (0.25–0.71)	0.39 (0.23–0.67)
P for trend			0.005	0.003
Sleeping time as a continuous variable (1 hour/day increase)			0.79 (0.67–0.94)	0.78 (0.66–0.93)

Hyperglycemia was defined as more than the 90^th^ percentiles of fasting glucose.

*Adjusted for maternal age, pre-pregnancy BMI, weight gain during pregnancy, education, occupation, family income, and child age, sex, gestational age, and mode of infant feeding.

†Adjusted for variables in Model 1 and also indoor activity, outdoor activity, TV watching time, and sleeping time, other than the variable in the analyses.
